# Heterogeneity in Primary Productivity Influences Competitive Interactions between Red Deer and Alpine Chamois

**DOI:** 10.1371/journal.pone.0146458

**Published:** 2016-01-29

**Authors:** Pia Anderwald, Rudolf M. Haller, Flurin Filli

**Affiliations:** Swiss National Park, Chastè Planta-Wildenberg, 7530 Zernez, Switzerland; University of Sassari, ITALY

## Abstract

Habitat heterogeneity can promote coexistence between herbivores of different body size limited to different extents by resource quantity and quality. Red deer (*Cervus elaphus*) are known as superior competitors to smaller species with similar diets. We compared competitive interactions and habitat use between red deer and Alpine chamois (*Rupicapra rupicapra*) in two adjacent valleys in a strictly protected area in the Central Alps. Red deer density was higher in the valley with higher primary productivity. Only here was horn growth in kid and yearling chamois (as a measure for body condition) negatively correlated with red deer population size, suggesting interspecific competition, and chamois selected meadows with steeper slopes and lower productivity than available on average. Conversely, red deer selected meadows of high productivity, particularly in the poorer area. As these were located mainly at lower elevations, this led to strong altitudinal segregation between the two species here. Local differences in interspecific competition thus coincided with differences in habitat preference and–segregation between areas. This suggests that spatial habitat and resource heterogeneity at the scale of adjacent valleys can provide competition refuges for competitively inferior mountain ungulates which differ from their superior competitor in their metabolic requirements.

## Introduction

Interspecific interactions between herbivores depend on resource availability, animal densities and the extent of overlap in resource use (e.g. [1]). Heterogeneity in habitat and resources can promote coexistence between species, especially if they differ in body size and feeding styles [2]. While spatial heterogeneity leads to aggregation of animals in resource hotspots, which can increase competition locally, a clumped distribution of the superior competitor can leave patches of lower densities or absence, i.e. ‘competition refuges’ for competitively inferior species [3, 4, 5]. Alpine environments represent highly heterogeneous habitats, with variation in resource quantity and quality e.g. between areas of different bedrock, over different altitudinal ranges, or dependent on the steepness and exposure of slopes [6]. Accordingly, interspecific interactions between herbivores would be expected to vary over the same fine spatial scales depending on habitat selection and specialization.

Many mountain ungulates show seasonal altitudinal migrations (e.g. [7, 8, 9, 10]), which can result in high grazing pressure on subalpine meadows by both resident and migratory species during summer. As the growing season is short at high elevations, foraging conditions during summer play an important role in determining growth, body condition and winter survival of large herbivores (e.g. [11,12,13, 14]), so that they are particularly sensitive to competition at this time of year (e.g.[15]). The period of horn growth in bovids coincides with the growing season, and horn growth in young animals is often correlated with an individual’s body condition [16, 17, 18]. Annual horn growth thus reflects habitat quality and resource availability [19, 20, 21, 22, 23], and due to this sensitivity to environmental conditions during the growing season is expected to also be affected by intra- and interspecific competition.

Among the native wild ungulates of Europe, red deer (*Cervus elaphus*) are known as superior competitors over smaller species such as roe deer (*Capreolus capreolus*) and Apennine chamois (*Rupicapra pyrenaica ornata*): for the two cervids, the presence of interspecific competition has been suggested based on inverse relationships in hunting records [24] and relative abundance [25, 26], habitat displacement of roe by red deer [27], and lower roe deer fawn body mass in the presence of red deer [28]. Moreover, local population declines of the threatened Apennine chamois in Italy have been linked to an increasing population size of red deer, with chamois suffering from decreases in diet quality and foraging efficiency, and resulting declines in kid survival, in areas with high red deer density [15, 29].

Although chamois morphology and diet are generally closer to that of a browser, both red deer and chamois are classified as mixed feeders [30, 31, 32], and diet overlap between the two species is high where they co-occur [15, 33, 34]. This strong overlap in diet also applies to the Swiss National Park in the Central Alps, where Alpine chamois (*Rupicapra rupicapra*) co-occur with high densities of red deer during summer. In the valley with highest red deer abundance, both horn growth in young (kid and yearling) chamois and the use of meadows (in favour of areas covered by scree) are negatively correlated with local red deer population size, suggesting interspecific competition with habitat displacement to poorer feeding areas [35]. However, as local red deer density varies between different valleys within the National Park, the clumped distribution of red deer may provide competition refuges for chamois. Differences in competitive interactions should in turn be linked to the physical characteristics of the local environment, which influence resource quantity and quality. The aim of this study was to a) establish whether competitive interactions between red deer and chamois differed between two valleys with different red deer density and primary productivity (approximated by the Normalized Difference Vegetation Index (NDVI); [36, 37]), and b) shed light on what role habitat segregation and -preferences at the local scale could play in driving these differences. We compared habitat suitability between the two valleys for both species based on group sizes (e.g. [38, 39]), determined the role of both chamois and red deer population size on horn growth in chamois, and then related local differences in competitive interactions to habitat segregation and -preferences within areas. We predicted that the more productive valley with higher red deer density represented a more favourable habitat for red deer, while chamois groups should be larger in the less productive valley due to lower degrees of interspecific competition. Furthermore, we expected chamois horn growth to be less affected by red deer population size in the less productive area with lower red deer density, but with intraspecific effects being more important here due to a comparatively higher chamois density. Habitat preferences within valleys were predicted to be driven mainly by productivity of meadows, with red deer preferring the more productive patches.

## Materials and Methods

Permission for this work was granted by the authorities of the Swiss National Park.

### Study area

**Fig 1:**
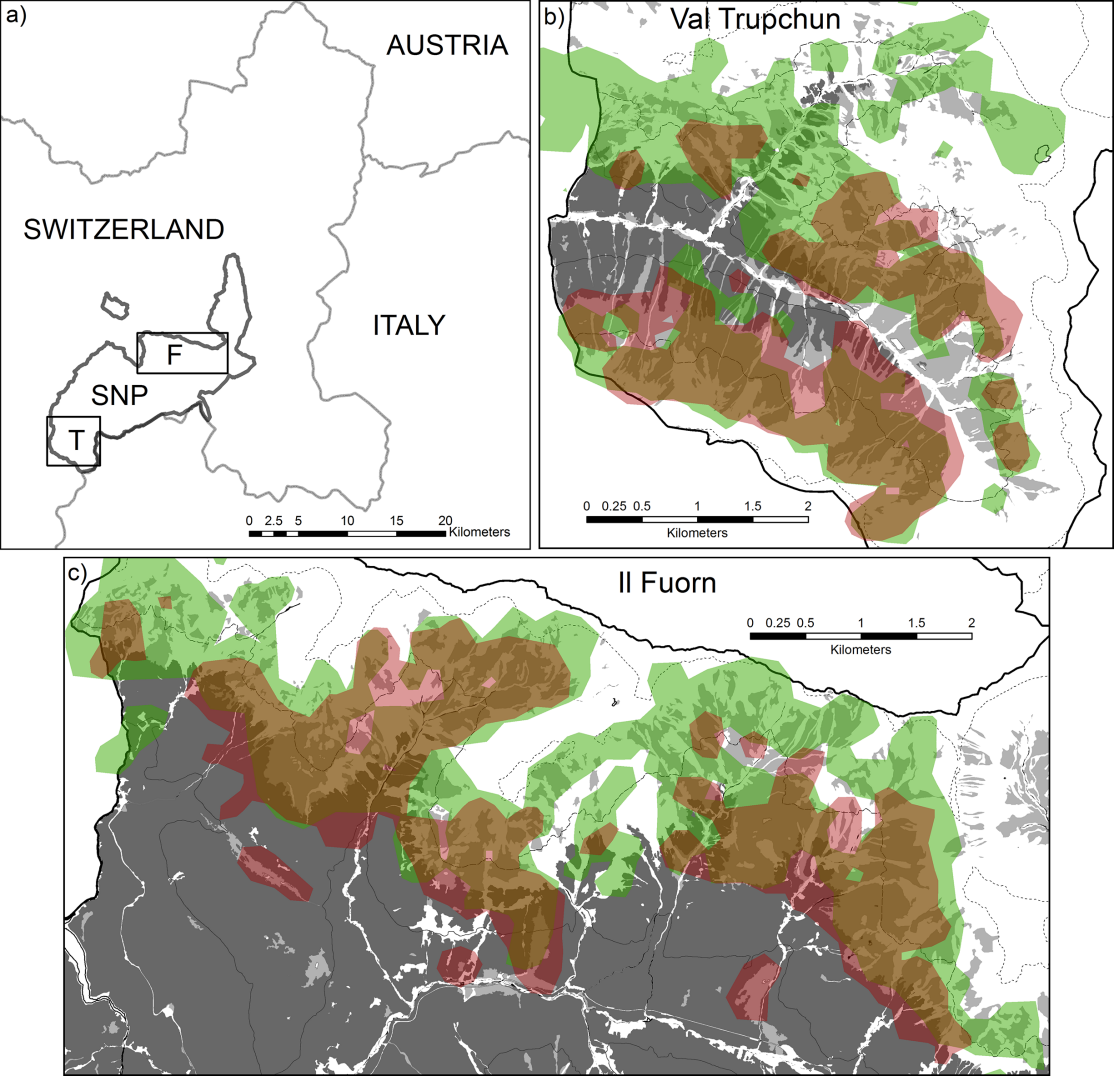
Location of the two ungulate study areas Val Trupchun and Il Fuorn in the Swiss National Park. a) SNP = Swiss National Park, T = Val Trupchun (enlarged in b)), F = Il Fuorn (enlarged in c)). The thick black line represents the National Park boundary, the thin gray line national boundaries between Switzerland, Italy and Austria. b, c) 95% Kernel densities (resolution = 30m; search radius = 250m) of chamois (green) and red deer (red; with areas of overlap between the two species indicated in brown), based on visual surveys during August 1997 to 2013. Dark grey shading in the background indicates conifer forest, light grey shading meadows, and white background bare soil (rock, scree or snow). The thin solid black line represents the 2000m, the dashed line the 2400m, and the dotted line the 2800m contours.

The study area was located in the Swiss National Park in the south-east of Switzerland in the Central Alps. Area 1 (Val Trupchun) was situated in the south-western, and area 2 (Il Fuorn) in the north central part of the park (Fig 1). Both valleys comprise elevations between 1800m and 2800m above sea level and consist predominantly of scree and rocks, alpine and subalpine grassland, and coniferous forest ([40]; Fig 1, Table 1). The underlying bedrock is mainly calcareous rock in Val Trupchun and dolomite in Il Fuorn. Although Val Trupchun is dominated by bare soil with steeper slopes than Il Fuorn, it contains proportionately more grassland, which is on average located at lower elevations and has consistently higher primary productivity (Table 1).

**Table 1:** Habitat characteristics of Val Trupchun and Il Fuorn. Habitat characteristics are based on 50m cells within convex hulls of chamois and red deer sightings in both areas (means ± std). Values for NDVI represent the lowest and highest yearly (August) means and standard deviations, respectively, over the study period.

	Trupchun	Il Fuorn
**Overall habitat:**		
**Elevation (m)**	2404 ± 257	2353 ± 278
**Slope (deg)**	36.1 ± 9.6	30.7 ± 11.4
**Ruggedness**	0.002 ± 0.004	0.002 ± 0.004
**Solar radiation (Wh/m**^**2**^**)**	39.6 ± 15.0	46.9 ± 14.6
**Habitat type (% cover):**		
**- Meadow**	27.5	17.3
**- Rock/scree**	61.2	36.9
**- Conifer forest**	10.7	44.9
**Meadows only:**		
**Elevation (m)**	2312 ± 175	2414 ± 184
**Slope (deg)**	35.2 ± 7.8	29.8 ± 9.5
**Ruggedness**	0.002 ± 0.003	0.001 ± 0.002
**Solar radiation (Wh/m**^**2**^**)**	40.0 ± 14.8	47.2 ± 13.4
**NDVI**	0.34 to 0.42 ± 0.20 to 0.28	0.21 to 0.28 ± 0.14 to 0.18

**Fig 2:**
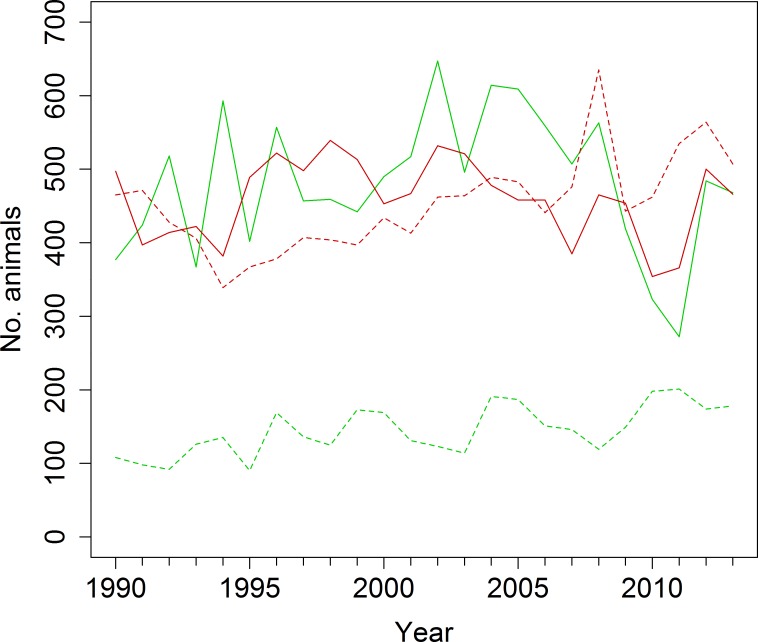
Annual counts of chamois and red deer in Val Trupchun and Il Fuorn 1990–2013. Green lines represent chamois, red lines red deer; solid lines represent Il Fuorn, broken lines Val Trupchun.

In both study areas, chamois and red deer co-occur only during the summer months. While chamois remain within the park or its vicinity year-round, most red deer spend the winter at lower elevations outside the park boundaries, where they are subjected to a culling programme [41]. Summer densities of the two species have reached up to 29 red deer and 9 chamois per km^2^ in Val Trupchun since 1990 (Fig 2). In addition, high ibex (*Capra ibex*) numbers occur on the valley slopes and can reach densities of up to 18 animals per km^2^. By contrast, ungulate densities are lower in Il Fuorn, with maximum numbers of 11 red deer, 13 chamois (Fig 2) and only one ibex per km^2^. Based on data from GPS/VHF-collared (n_chamois_ = 76, n_red deer_ = 58) and ear-tagged animals (n_chamois_ = 203, n_red deer_ = 76), respectively, exchange of individual chamois and red deer between the two study areas is limited. As ibex do not appear to affect chamois negatively through competition with respect to population trends or body condition in Val Trupchun [35], and the species’ density is negligible in Il Fuorn, ibex was excluded from the analysis.

Within the boundaries of the Swiss National Park, animals are protected from anthropogenic disturbance by a hunting ban and by visitors being strictly prohibited from leaving the footpaths or bringing dogs into the park. There have been no resident natural mammalian predators in the study area since the 19th century. The park is thus ideally suited to studying species interactions between free-living ungulates in the absence of natural predators or anthropogenic disturbance. The safety of the National Park also results in red deer using alpine pastures for feeding, ruminating and resting even during daytime instead of retreating to the forest during daylight hours as they tend to do where they are regularly disturbed by humans (e.g. [42, 43]). This high use of open areas by the animals increases their visibility and thus improves the accuracy of population counts and mapping their spatial distribution.

### Population counts

Annual counts of chamois and red deer population sizes (S1 Dataset) have been conducted by experienced park rangers during the second half of July (i.e. at maximum seasonal density of both species) since 1990. The two study areas were sub-divided into five (Val Trupchun) and seven (Il Fuorn) separate blocks, and counts conducted from the same fixed observation points each year, which were selected based on the optimal viewshed from each point. Observation points overlooked the opposite slope of the valley, so that animals remained undisturbed during the surveys. To standardize counts, they were carried out within a window of two weeks each year on days with optimal viewing conditions. Double-counting of animals close to the boundaries of adjacent blocks was avoided by noting the time of the sightings, their exact locations and group compositions, and subsequent comparison of these notes between rangers immediately after the surveys (see also [44]). Counts were conducted twice within the two-week period in July, and the higher number of animals detected between surveys was taken as the minimum local population estimate.

### Group size

It is expected that animals occur in larger groups in more productive food patches (e.g. [38, 39]). We therefore compared group sizes on meadows (S2 Dataset; based on surveys mapping the spatial distribution of the animals; see section on *Habitat Use*) for both species between the two study areas using a Mann-Whitney-U test.

### Horn growth

Horn growth of young chamois (S3 Dataset) was used as a proxy for body condition [16, 17, 18]. All skulls of chamois found dead within the two study areas have been collected and catalogued with associated metadata (location, date, gender and age of animal, condition of the skull and horns etc.). Data on horn growth were additionally available from tagging protocols of animals captured for ear-tagging and VHF/GPS-collaring. All capture, handling and tagging methods were approved by the veterinary office of the canton Grison, Switzerland. Annual growth sections of horns of individuals ≥ 2 years old were measured along the posterior end of the horns using a tape measure. The analysis was restricted to the kid and yearling years combined (i.e. the length from the tip of the horns to the end of the second annulus) for chamois born in 1990 or later. This resulted in sample sizes of 76 skulls from animals found dead, but fresh enough for determination of the year of death and with their horns in good condition, and 31 measured skulls from tagging protocols (n_Trupchun_ = 55, n_Fuorn_ = 52). Horn growth was expressed as the average horn length between left and right sides.

In order to test for differences in chamois horn growth between the two valleys and distinguish between influences at the regional (e.g. seasonal climatic conditions) and local scale (e.g. animal densities per valley), we performed two linear regressions. The regional model included the amount of precipitation (mm) during the growing season (mid May to mid September) over the kid and yearling years combined, area and sex as explanatory variables. The amount of precipitation during the growing season was used as a measure for climatic variation determining seasonal food availability and was calculated from climate data recorded at the weather station Buffalora [45], located at 1968 m a.s.l. (46.65 N, 10.27 E) at a distance of ca. 1.5 km from Il Fuorn and ca. 13 km from Val Trupchun. In addition to sex and precipitation, the local model for Il Fuorn included the (local) mean number of chamois and red deer between the kid and yearling years as explanatory variables, along with first order interaction terms between sex and the three continuous parameters. The local model for Il Fuorn thus had the same structure as previously described for Val Trupchun [35], but with precipitation and its interaction with sex included in addition. We repeated the local model for Val Trupchun from [35] with these parameters included to enable a direct comparison. All continuous variables were standardized to enable comparison between regression coefficients. Model selection was based on the Akaike Information Criterion corrected for small sample sizes (AICc; [46]). As AIC measures tend to favour overly complex models (e.g. [47, 48]), we selected the most parsimonious model within ∆AICc ≤ 2 considering all possible predictor combinations. However, the relative importance of each parameter was also assessed by calculating predictor weights based on model weights taking all candidate models into account [46]. Residual patterns were checked for normality, homogeneity of variances and absence of leverage values. Analyses were conducted in R version 3.0.2 [49], using the AICcmodavg package [50] to calculate AICc.

### Habitat use

The summer spatial distribution of chamois and red deer in both study areas was mapped by National Park rangers during one morning each in the first half of August every year between 1997 and 2013. Although such a sampling procedure only provides ‘snapshots’ of the animals’ distribution, the data can serve as a baseline for making inferences on habitat use thanks to the highly traditional use of seasonal feeding grounds, especially by red deer. The same method has been applied successfully elsewhere to assess interspecific habitat associations in alpine ungulates (e.g. [51]). The study areas were divided into the same sub-areas as for the population counts in July, and each ranger noted the locations, size and composition of all ungulate groups (distance between individuals ≤100 m) within his sub-area (rotated between rangers on a regular basis) on a printed map at a resolution of 1:25,000 (http://www.swisstopo.admin.ch). Animals were mapped with an average accuracy of 30 m [52]. To standardize survey conditions, the same observation points were used over the entire 17-year period, and surveys were conducted only in optimal viewing conditions (i.e. no precipitation or fog).

All sighting locations of chamois and red deer were digitized and differences in animal group sizes accounted for by multiplying each sighting record with its associated environmental variables by the number of individuals in the group. Only adult animals were taken into consideration to avoid pseudo-replicating sighting locations by kids and yearlings following their mothers. The analysis was restricted to individuals seen on alpine and subalpine meadows, where both species predominantly feed within the park during summer (S4 Dataset). Environmental variables included the following:

- Elevation (m), slope (degrees) and solar radiation (WH/m^2^; at 10am local time on 7^th^ August) from a digital elevation model of 4 x 4 m resolution, based on digital photogrammetry [53].
- Terrain ruggedness: calculated after [54] and based on 9 x 9 cells of the 4 x 4 m elevation grid.
- Normalized Difference Vegetation Index (NDVI): based on Landsat-5 TM and Landsat-7 ETM+ level 1 satellite data (spatial resolution: 30 m, temporal resolution: 16 days). Downloaded from http://glovis.usgs.gov and processed in ArcGIS according to http://ibis.colostate.edu/WebContent/WS/ColoradoView/TutorialsDownloads/CO_RS_Tutorial8.pdf and CO_RS_Tutorial10.pdf, respectively, with radiometric calibration coefficients obtained from [55].

All calculations were performed within ArcGIS Version 10.1 using the CH1903+_LV95 projection, with distance units set to metres.

The availability of high-quality satellite data over the survey period in August for the calculation of NDVI values restricted the analysis to the years 2001, 2003–06, 2008–09 and 2013 for Il Fuorn, and to 2001, 2003–06, 2008–11 and 2013 for Val Trupchun.

All environmental parameters were checked for collinearity for both study areas using Spearman correlation coefficients. Generalized Linear Mixed Models (GLMM) with a binomial error structure and ‘Year’ as a random factor were then applied for sightings positions of chamois (coded as 1) vs. red deer (coded as 0) in the lme4 library [56] in R [49]. Due to initial convergence problems for Il Fuorn with untransformed explanatory variables, all environmental parameters were standardized for inclusion in the models for both study areas. Model selection was performed according to AICc and predictor weights [46] in the same manner as for horn growth.

We then compared habitat use with available habitat with respect to the most important environmental parameters separating chamois and red deer in both study areas (S5 Dataset). A convex hull was constructed around all chamois and red deer sightings in Il Fuorn and Val Trupchun, respectively, a 50m grid overlaid, and environmental variables extracted for the centre of each grid cell, where the centre was located on a meadow. Sightings were linked to these grid cell centres via a spatial join. Following the concept of marginality and specialization [57], we then calculated the deviation from the mean (mean_used_−mean_available_) and ratio of standard deviations (SD_used_ / SD_available_) between used and available habitat for each environmental parameter. Measures for available NDVI were calculated based on values over all grid cells per area over all years.

## Results

### Group size

**Fig 3:**
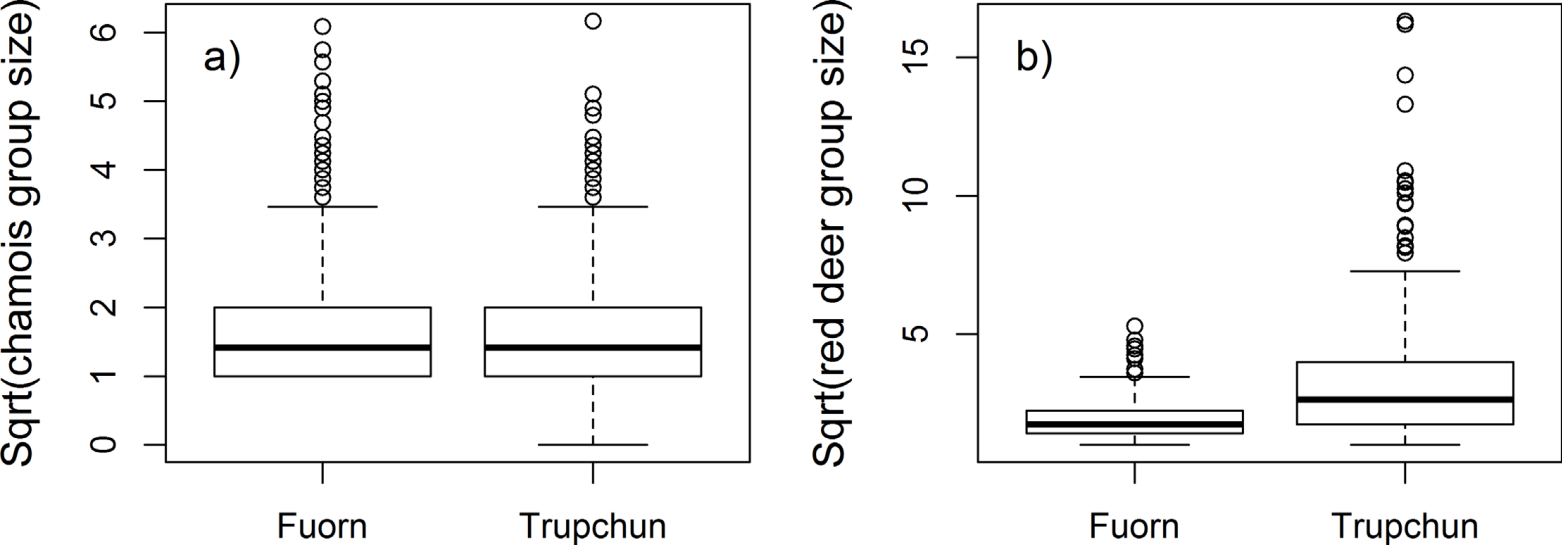
Group sizes of chamois (a) and red deer (b) on meadows in Il Fuorn and Val Trupchun.

While chamois group size on meadows did not differ between the two areas (U = 71260, p = 0.211; n_Trupchun_ = 321, n_Fuorn_ = 467 groups; Fig 3A), red deer showed significantly higher group sizes on meadows in Val Trupchun than in Il Fuorn (U = 34797, p < 0.001; n_Trupchun_ = 287, n_Fuorn_ = 437 groups: Fig 3B).

### Horn growth

**Fig 4:**
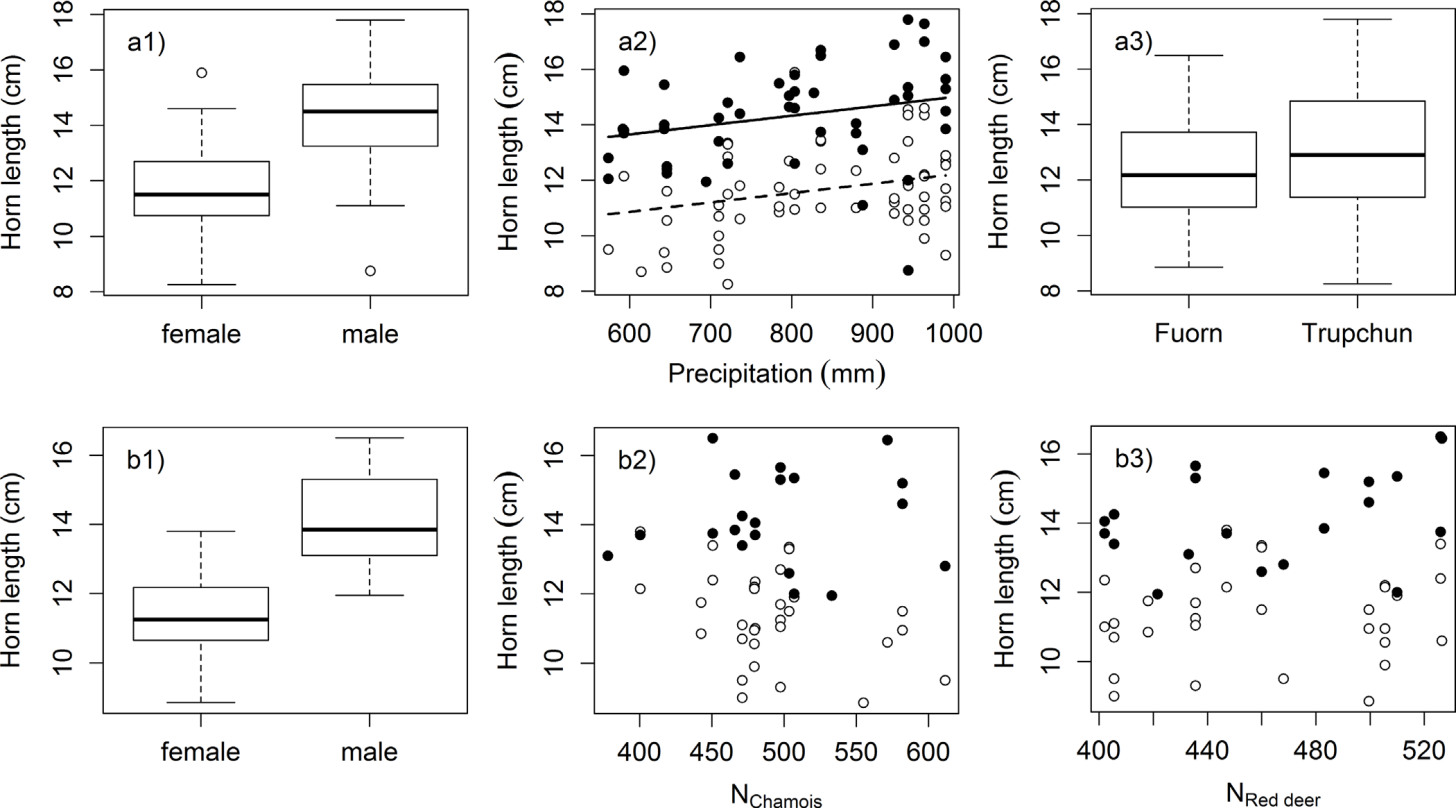
Horn growth. Chamois horn growth during the kid and yearling years combined vs. explanatory variables in regional (Val Trupchun and Il Fuorn combined; a1-3) and local (Il Fuorn only; b1-3) linear models. Open symbols in scatterplots represent females, closed symbols males. Plots are shown for all parameters with predictor weights > 50%, but regression lines are only included for continuous variables retained in the most parsimonious model within ∆AICc ≤ 2.

The two highest-ranked regional linear models for horn growth in kid and yearling chamois (Val Trupchun and Il Fuorn combined) included area, sex and precipitation, and only sex and precipitation, respectively. Both models were well supported compared to lower-ranked models, but little difference was detected between them (∆AICc = 0.53; Table 2). We therefore selected the more parsimonious model, including only sex and precipitation. This was also supported by the relative importance of parameters based on predictor weights over all models, which suggested that sex had a 95% probability of being a component of the best model, precipitation a 97% probability, but area only a 57% probability (P). According to this model, male chamois grew longer horns than females (estimate = 2.792, SE = 0.323) and summer precipitation had a positive influence on horn growth (estimate = 0.445, SE = 0.161; Fig 4A).

**Table 2:** Horn growth. AICc-based ranking of candidate linear models explaining variation in horn growth in kid and yearling chamois across the two study areas (regional model) and in Il Fuorn (local model). Only the five highest-ranked models are shown. X indicates that the parameter was included in the model, a gap means that it was considered in the global model but excluded from the specific model, and ‘n.a.’ indicates that the parameter was not applicable for the regional or local model, respectively.

	Area	Sex	Precipitation	No. chamois	No. red deer	Sex * Precipitation	Sex * No. chamois	Sex * No. red deer	ΔAICc	AICc weight
**Regional model**	X	X	X	n.a.	n.a.	n.a.	n.a.	n.a.	0.00	0.52
		X	X	n.a.	n.a.	n.a.	n.a.	n.a.	0.53	0.40
	X	X		n.a.	n.a.	n.a.	n.a.	n.a.	4.89	0.05
		X		n.a.	n.a.	n.a.	n.a.	n.a.	5.95	0.03
	X		X	n.a.	n.a.	n.a.	n.a.	n.a.	55.32	0.00
**Il Fuorn model**	n.a.	X		X	X		X		0.00	0.19
	n.a.	X		X	X				0.84	0.12
	n.a.	X							1.30	0.10
	n.a.	X		X			X		1.60	0.08
	n.a.	X			X				1.75	0.08

The five highest-ranked models for chamois horn growth in Il Fuorn all had low support, with the most parsimonious model including only sex showing a difference of ∆AICc = 1.3 compared to the highest-ranked model (Table 2). Predictor weights based on all candidate models highlighted the high importance of sex (P = 100%; with males growing longer horns than females: estimate = 2.750, SE = 0.382; Fig 4B1) compared to other variables (P_Number of chamois_ = 72% (Fig 4B2), P_Number of red deer_ = 66% (Fig 4B3), P_Sex * Number of chamois_ = 43%, P_Precipitation_ = 24%, P_Sex * Number of red deer_ = 15%, and P_Sex * Precipitation_ = 5%). The comparatively high probabilities for number of chamois and number of red deer indicated that these parameters included in the second highest-ranked model (Table 2) should not be discounted as components of the best model, suggesting a negative effect of chamois population size (estimate = -0.345, SE = 0.193) and a positive relationship with red deer population size (estimate = 0.359, SE = 0.193). However, the very wide confidence limits for both variables confirmed their limited contribution (see also Fig 4B2 and 4B3).

This was in contrast to results from Val Trupchun, where horn growth in young chamois according to the same model structure had been negatively related to red deer population size (estimate = -1.031, SE = 0.235), while also being dependent on sex (estimate = 3.004, SE = 0.466), but independent of chamois population size [35]. Predictor weights indicated strong support for both parameters (P_Sex_ = P_Number of red deer_ = 100%) by comparison to all other variables (P_Sex * Number of red deer_ = 35%, P_Precipitation_ = 33%, P_Number of chamois_ = 32%, P_Sex * Precipitation_ = 18%, and P_Sex * Number of chamois_ = 6%),.

### Habitat use

While there were no sightings of either species below 2000m in Val Trupchun during August (Fig 1B), chamois and red deer sightings on meadows in Il Fuorn occurred between elevations from 1800m to 2800m, with sightings below 2000m dominated by female red deer on former forest pastures (Fig 1C).

**Fig 5:**
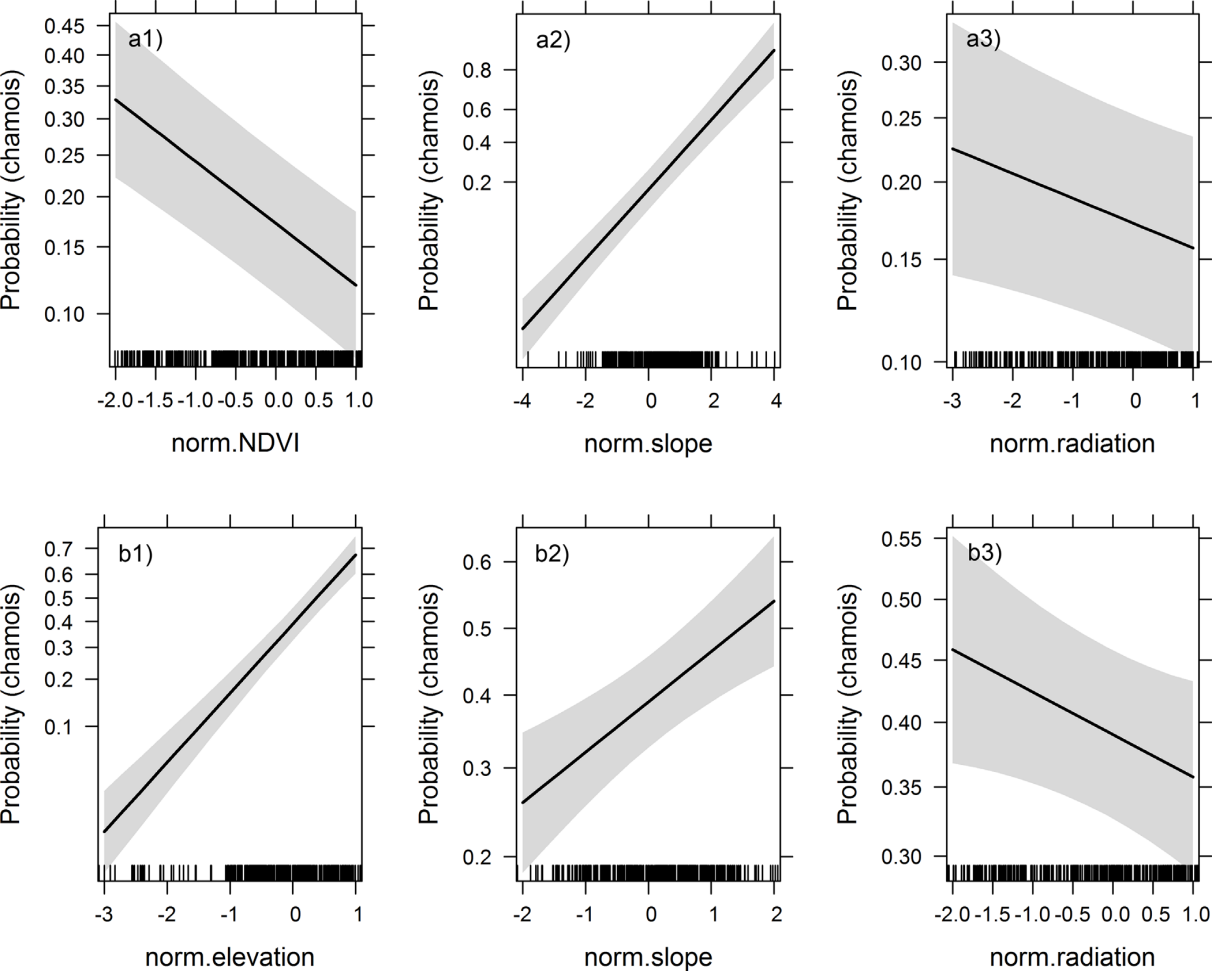
Habitat use. Effects of parameters (with standard errors in gray) included in most parsimonious GLMM’s within ∆AICc ≤ 2 for use of meadows by adult chamois (coded as 1) vs. red deer (coded as 0) in a) Val Trupchun and b) Il Fuorn. The y-axes are on a logarithmic scale.

Val Trupchun: Spearman correlations between environmental parameters were weak for Val Trupchun (|rho| ≤ 0.4). The highest-ranked model differentiating chamois from red deer habitat included slope, NDVI and radiation and was the most parsimonious model within ∆AICc ≤ 2 (Table 3; P_Slope_ = 100%, P_NDVI_ = 100%, P_Radiation_ = 75%, P_Elevation_ = 34%, and P_Ruggedness_ = 27%). The probability of encountering chamois rather than red deer increased with increasing slope (estimate = 0.859, SE = 0.067) and decreasing NDVI (estimate = -0.428, SE = 0.062) and solar radiation, although the confidence limit for this latter parameter was wide (estimate = -0.112, SE = 0.051; Fig 5A).

**Table 3:** Habitat use. AICc-based ranking of candidate GLMM’s explaining differences in habitat use between chamois (coded as 1) and red deer (coded as 0) in Val Trupchun and Il Fuorn. Only the five highest-ranked models are shown.

	Radiation	Elevation	Slope	Ruggedness	NDVI	ΔAICc	AICc weight
**Val Trupchun**	X		X		X	0.00	0.38
	X	X	X		X	1.65	0.17
	X		X	X	X	1.95	0.14
			X		X	2.84	0.09
		X	X		X	3.12	0.08
**Il Fuorn**	X	X	X			0.00	0.32
	X	X	X	X		0.99	0.20
	X	X	X		X	1.52	0.15
		X	X			2.16	0.11
	X	X	X	X	X	2.44	0.10

Il Fuorn: There was a moderate negative correlation (rho = -0.566) between elevation and NDVI for Il Fuorn, but models in which elevation was replaced by NDVI ranked low (∆AICc > 10). Correlations between all other variables were weak (|rho| ≤ 0.284). The highest-ranked model was also the most parsimonious within ∆AICc ≤ 2 (Table 3) and included elevation (P = 100%), slope (P = 100%) and radiation (P = 77%). Ruggedness (P = 37%) and NDVI (P = 31%) were less important by comparison. In the latter case, this was probably caused by the correlation between NDVI and elevation. The probability of encountering chamois as opposed to red deer increased with increasing elevation (estimate = 1.185, SE = 0.104), increasing slope (estimate = 0.306, SE = 0.077) and decreasing solar radiation, although the confidence limit for the latter was wide (estimate = -0.140, SE = 0.069; Fig 5B).

There was greater interannual variation in the distribution of the two species relative to each other in Val Trupchun (std_Year_ = 0.766) than in Il Fuorn (std_Year_ = 0.341).

When comparing actual habitat use by the two species with available habitat, red deer showed a preference for meadows with higher mean NDVI values than available on average in both study areas, whereas habitat selection by chamois was the converse (Table 4). This selection for meadows with lower NDVI values by chamois was stronger in Val Trupchun, the area with higher primary productivity, whereas red deer selected more strongly for meadows with high NDVI values in Il Fuorn, the less productive area.

**Table 4:** Comparison between used and available habitat for chamois and red deer in Val Trupchun and Il Fuorn with respect to deviation from the mean and specialization (SD_used_ / SD_available_). All values are in %, i.e. means are expressed as fractions of the total available range, with absolute differences given in brackets.

	Chamois		Red deer		Chamois		Red deer	
	Mean (%)		Mean (%)		SD		SD	
	Trupchun	Il Fuorn	Trupchun	Il Fuorn	Trupchun	Il Fuorn	Trupchun	Il Fuorn
**Elevation**	+3.6	+3.0	+2.5	-7.4	86.5	93.0	82.3	104.3
	(35.9m)	(32.5m)	(25.5m)	(79.6m)				
**Slope**	+5.0	-0.1	+0.6	-4.4	80.1	84.1	78.9	100.6
	(3.0deg)	(0.05deg)	(0.4deg)	(3.0deg)				
**Radiation**	+2.6	-0.1	+10.0	+2.1	118.0	97.4	88.6	89.0
	(1.6Wh/m^2^)	(0.04Wh/m^2^)	(6.1Wh/m^2^)	(1.2Wh/m^2^)				
**NDVI**	-5.9	-1.5	+2.9	+5.0	93.6	89.0	93.7	105.9
	(0.05)	(0.01)	(0.03)	(0.04)				

In Val Trupchun, where both species selected meadows at higher than average elevations, chamois showed a preference for steeper slopes. Such a preference was absent in Il Fuorn, where red deer selected lower and chamois higher elevations than available on average, resulting in strong segregation by elevation (see above).

Specialization was strongest with respect to slope for both red deer and chamois in Val Trupchun (79% and 80%, respectively; Table 4). Overall, red deer showed stronger specialization in Val Trupchun than in Il Fuorn, whereas there was no difference in specialization between areas for chamois.

## Discussion

When species overlap in their resource use to a large extent as do red deer and chamois [15, 33, 34], the inferior competitor (in this case chamois [15, 29]) is expected to perform better in an area with lower densities of the superior competitor, but only if the habitat quality of both areas is comparable. Our two study areas were contrasted by high primary productivity coinciding with high red deer densities (Val Trupchun), and lower primary productivity combined with lower red deer densities, respectively (Il Fuorn). Based on negative effects of red deer population size on horn growth in young chamois as a measure for body condition, interspecific competition affected chamois only in Val Trupchun [35], but not in Il Fuorn (Fig 4). As expected, this difference in competitive interactions between the two study areas was consistent with their respective habitat suitability for red deer (Fig 3) and resulting differences in red deer densities, but also seemed to be linked to red deer habitat preferences within areas (Table 4). Based on significantly larger group sizes (Fig 3B), Val Trupchun with its consistently higher primary productivity (measured as NDVI; Table 1) represented a more suitable habitat for red deer than Il Fuorn. This did not apply to chamois, which showed no difference in group size on meadows between the two areas. By contrast to red deer, the two study areas thus seemed to represent habitats of similar quality for chamois.

Plant biomass tends to decrease with increasing elevation (e.g. [58]), and sites are not grazed below a certain productivity threshold [59]. As larger herbivores are more constrained by diet quantity than smaller species [31], this threshold is likely to be lower for red deer than for chamois. Indeed, red deer tend to exploit food patches of both relatively high forage quantity and quality (e.g. [60, 61]). Accordingly, they selected meadows with higher than average NDVI, i.e. high primary productivity, in both study areas, but particularly in Il Fuorn, the less productive area (Table 4). Here, meadows with high NDVI were located mainly at lower elevations on former forest clearings, resulting in moderate negative correlations between NDVI and elevation (average rho = -0.495 between years; range: -0.6 ≤ rho ≤ -0.375). This was also confirmed by maps of biomass distribution on meadows derived from airborne imaging spectroscopy data combined with ground referencing in 2010 [52]. Meadows at higher elevations, which were preferred by chamois (Table 4), were thus probably not productive enough to sustain red deer over longer periods of time during the summer, which led to a strong segregation by elevation between the two species (Fig 5B1). This spatial heterogeneity in resources may have provided ‘escape areas’ or competition refuges [3, 4] for the smaller species, which is more constrained by diet quality than quantity (e.g. [62]).

In Val Trupchun, the area with high primary productivity, the correlation between NDVI and elevation on meadows was weaker than in Il Fuorn (average rho = -0.327 between years; range: -0.407 ≤ rho ≤ -0.217; see also biomass map in Fig 5A in [63]). For red deer, the selection for meadows with high solar radiation and high NDVI values coincided with a positive selection for elevation, which was similar to the altitudinal selection by chamois (Table 4) and thus led to elevation playing only a minor role in habitat segregation between the two species here. On the other hand, slope was an important parameter in separating the habitats of chamois and red deer in both areas (Table 3). In Il Fuorn, this was mainly caused by a negative selection for steeper slopes by red deer, in Val Trupchun by a positive selection for steeper slopes by chamois (Table 4). Given that slopes in Val Trupchun were steeper on average than in Il Fuorn (Table 1) and chamois showed no selection for slope and little selection for NDVI in the latter area with lower red deer density, the preference of meadows with steeper slopes and lower NDVI in Val Trupchun could indicate spatial avoidance by chamois of the superior competitor. Steep and less productive areas may thus have provided ‘refuge areas’ in Val Trupchun. The decreased use of meadows in favour of scree slopes by chamois with increasing red deer population size [35] may further support this interpretation. Consistent with dynamic spatial partitioning [64], chamois often grazed the same meadows in autumn which had formerly been occupied by red deer during the summer (personal observations), after the dominant competitor had left Val Trupchun to spend the winter at lower elevations outside the park boundaries.

Maximum red deer densities in our two study areas were only slightly below (Il Fuorn: up to 11 red deer / km^2^) and twice as high (Val Trupchun: 29 individuals / km^2^), respectively, as red deer densities in [15]’s study in the Italian Apennine (14 animals / km^2^). Yet, by contrast to their work, we found no evidence for interspecific competition between red deer and chamois in Il Fuorn, and an effect of red deer numbers only on horn growth, but not population growth rate [35], of chamois in Val Trupchun. Based on the fine-scale differences in interspecific interactions between the two adjacent valleys examined here, we suggest that this discrepancy between the Apennine and our two study areas may be linked to differences in habitat, i.e. mainly the availability of refuge areas of lower primary productivity for chamois, which appeared to be unattractive to red deer. The high spatial habitat and resource heterogeneity in Il Fuorn and Val Trupchun, both located at elevations over 400m higher than [15]’s study area, thus appears to promote the coexistence of these two herbivores with different metabolic requirements, of which one is more limited by resource quantity (red deer) and the other by resource quality (chamois). However, local interactions between the two species may be altered by climate change over the longer term. At a continental scale, mountain plant communities above the treeline are gradually being transformed, with cold-adapted species declining and warm-adapted species increasing [65]. Moreover, correlation models for grasslands in the Swiss Alps predict that increasing temperatures will lead to earlier snow melt in spring and thus earlier plant growth with increasing plant height and above-ground biomass (by ca. 45% by the end of the century [66]). An increase in both the length of the growing season and primary productivity on alpine meadows may result in red deer arriving on their summer feeding grounds earlier, staying longer and gradually expanding their summer ranges to higher elevations, which may reduce the availability of refuge areas for chamois in the long term.

## Supporting Information

S1 Dataset Annual counts of chamois and red deer population sizes in Val Trupchun and Il Fuorn. (XLSX)

S2 Dataset Group sizes of chamois and red deer in Val Trupchun and Il Fuorn. (XLSX)

S3 Dataset Horn growth of kid and yearling chamois. (XLSX)

S4 Dataset Environmental parameters associated with chamois and red deer sightings in Val Trupchun and Il Fuorn. (XLSX)

S5 Dataset Available habitat in Val Trupchun and Il Fuorn based on 50m grid cells. (XLSX)

## References

[1] De Boer WF, Prins HHT (1990) Large herbivores that strive mightily but eat and drink as friends. Oecologia 82(2): 264–274. 28312674 10.1007/BF00323544

[2] Owen-Smith N (2002) Adaptive herbivore ecology Cambridge University Press, Cambridge, UK.

[3] Begon M, Harper JL, Townsend CR (1996) Ecology: individuals, populations and communities Third edition Blackwell Science Ltd., Oxford, UK.

[4] Durant SM (1998) Competition refuges and coexistence: an example from Serengeti carnivores. J Anim Ecol 67: 370–386.

[5] Hobbs NT, Gordon IJ (2010) How does landscape heterogeneity shape dynamics of large herbivore populations? In: Owen-Smith N, editor. Dynamics of large herbivore populations in changing environments: towards appropriate models. Blackwell Publishing Ltd., Oxford, UK pp. 141–164.

[6] Nagy L, Grabherr G (2009) The biology of alpine habitats Oxford University Press, Oxford, UK.

[7] Oosenbrug SM, Theberge JB (1980) Altitudinal movements and summer habitat preferences of woodland caribou in the Kluane ranges, Yukon territory. Arctic 33: 59–72.

[8] Mysterud A (1999) Seasonal migration pattern and home range of roe deer (*Capreolus capreolus*) in an altitudinal gradient in southern Norway. J Zool 247: 479–486.

[9] White PJ, Proffitt KM, Mech LD, Evans SB, Cunningham JA, Hamlin KL (2010) Migration of northern Yellowstone elk: implications of spatial structuring. J Mammal 91(4): 827–837.

[10] Mysterud A, Loe LE, Zimmermann B, Bischof R, Veiberg V, Meisingset E (2011) Partial migration in expanding red deer populations at northern latitudes–a role for density dependence? Oikos 120: 1817–1825.

[11] Côté SD, Festa-Bianchet M (2001) Birthdate, mass and survival in mountain goat kids: effects of maternal characteristics and forage quality. Oecologia 127: 230–238. 10.1007/s004420000584 24577654

[12] Herfindal I, Sæther B-E, Solberg EJ, Andersen R, Høgda KA (2006) Population characteristics predict responses in moose body mass to temporal variation in the environment. J Anim Ecol 75: 1110–1118. 16922846 10.1111/j.1365-2656.2006.01138.x

[13] Pettorelli N, Pelletier F, von Hardenberg A, Festa-Bianchet M, Côté SD (2007) Early onset of vegetation growth vs. rapid green-up: impacts on juvenile mountain ungulates. Ecology 88(2): 381–390. 17479756 10.1890/06-0875

[14] Cook RC, Cook JG, Vales DJ, Johnson BK, McCorquodale SM, Shipley LA, et al (2013) Regional and seasonal patterns of nutritional condition and reproduction in elk. Wildl Monogr 184: 1–45.

[15] Lovari S, Ferretti F, Corazza M, Minder I, Troiani N, Ferrari C, et al (2014) Unexpected consequences of reintroductions: competition between reintroduced red deer and Apennine chamois. Anim Conserv 17(4): 359–370.

[16] Côté SD, Festa-Bianchet M, Smith KG (1998) Horn growth in mountain goats (*Oreamnos americanus*). J Mammal 79: 406–414.

[17] Rughetti M, Festa-Bianchet M (2010) Compensatory growth limits opportunities for artificial selection in alpine chamois. J Wildl Manage 74: 1024–1029.

[18] Rughetti M, Festa-Bianchet M (2011) Effects of early horn growth on reproduction and hunting mortality in female chamois. J Anim Ecol 80: 438–447. 10.1111/j.1365-2656.2010.01773.x 21073455

[19] Jorgenson JT, Festa-Bianchet M, Wishart WD (1998) Effects of population density on horn development in bighorn rams. J Wildl Manage 62: 1011–1020.

[20] Toïgo C, Gaillard J-M, Michallet J (1999) Cohort affects growth of males but not females in alpine ibex (*Capra ibex ibex*). J Mammal 80(3): 1021–1027.

[21] Giacometti M, Willing R, Defila C (2002) Ambient temperature in spring affects horn growth in male alpine ibexes. J Mammal 83: 245–251.

[22] Festa-Bianchet M, Coltman DW, Turelli L, Jorgenson JT (2004) Relative allocation to horn and body growth in bighorn rams varies with resource availability. Behav Ecol 15: 305–312.

[23] Chirichella R, Ciuti S, Grignolio S, Rocca M, Apollonio M (2013) The role of geological substrate for horn growth in ungulates: a case study on Alpine chamois. Evol Ecol 27(1): 145–163.

[24] Vladyshevskii DV (1968) Factors affecting the numbers of European roe deer (*Capreolus capreolus*). Zool Zh 47: 438–443.

[25] Latham J, Staines BW, Gorman ML (1996) The relative densities of red (*Cervus elaphus*) and roe (*Capreolus capreolus*) deer and their relationship in Scottish forests. J Zool 240: 285–299.

[26] Latham J, Staines BW, Gorman ML (1997) Correlations of red (*Cervus elaphus*) and roe (*Capreolus capreolus*) deer densities in Scottish forests with environmental variables. J Zool 242: 681–704.

[27] Danilkin AA (1996) Behavioural Ecology of Siberian and European Roe Deer. Chapman & Hall, London, UK.

[28] Richard E, Gaillard J-M, Saïd S, Hamann J-L, Klein F (2010) High red deer density depresses body mass of roe deer fawns. Oecologia 163: 91–97. 10.1007/s00442-009-1538-z 20033821

[29] Ferretti F, Corazza M, Campana I, Pietrocini V, Brunetti C, Scornavacca D, Lovari S (2015) Competition between wild herbivores: reintroduced red deer and Apennine chamois. Behav Ecol 26: 550–559.

[30] Gordon IJ, Illius AW (1988) Incisor arcade structure and diet selection in ruminants. Funct Ecol 2: 15–22.

[31] Hofmann RR (1989) Evolutionary steps of ecophysiological adaptation and diversification of ruminants: a comparative view of their digestive system. Oecologia 78: 443–457. 28312172 10.1007/BF00378733

[32] Pérez-Barbería FJ, Gordon IJ, Nores C (2001) Evolutionary transitions among feeding styles and habitats in ungulates. Evol Ecol Res 3: 221–230.

[33] Schröder J, Schröder W (1984) Niche breadth and overlap in red deer *Cervus elaphus*, roe deer *Capreolus capreolus* and chamois *Rupicapra rupicapra*. Ann Zool Fenn 172: 85–86.

[34] Bertolino S, di Montezemolo NC, Bassano B (2009) Food-niche relationships within a guild of Alpine ungulates including an introduced species. J Zool 277: 63–69.

[35] Anderwald P, Herfindal I, Haller RM, Risch AC, Schütz M, Schweiger AK, Filli F (2015) Influence of migratory ungulate management on competitive interactions with resident species in a protected area. Ecosphere 6(11): 228.

[36] Pettorelli N, Vik JO, Mysterud A, Gaillard J-M, Tucker CJ, Stenseth NC (2005) Using the satellite-derived NDVI to assess ecological responses to environmental change. Trends Ecol Evol 20(9): 503–510. 16701427 10.1016/j.tree.2005.05.011

[37] Pettorelli N, Ryan S, Mueller T, Bunnefeld N, Jędrzejewska B, Lima M, Kausrud K (2011) The Normalized Difference Vegetation Index (NDVI): unforeseen successes in animal ecology. Clim Res 46: 15–27.

[38] Winnie JA Jr, Cross P, Getz W (2008) Habitat quality and heterogeneity influence distribution and behavior in African buffalo (*Syncerus caffer*). Ecology 89(5): 1457–1468. 18543637 10.1890/07-0772.1

[39] Mbora DN, Wieczkowski J, Munene E (2009) Links between habitat degradation, and social group size, ranging, fecundity, and parasite prevalence in the Tana River mangabey (*Cercocebus galeritus*). Am J Phys Anthropol 140(3): 562–571. 10.1002/ajpa.21113 19544575

[40] Zoller H (1995) Vegetationskarte des Schweizerischen Nationalparks. Nationalpark Forschung Schweiz 85: 1–108.

[41] Jenny H, Filli F (2014) Wildforschung erarbeitet Grundlagen für Schutz und Jagd In: Baur B, Scheurer T, editors. Wissen schaffen. 100 Jahre Forschung im Schweizerischen Nationalpark. Nationalpark-Forschung in der Schweiz 100/I Haupt Verlag, Bern, Switzerland pp. 235–267.

[42] Náhlik A, Sándor G, Tari T, Király G (2009) Space use and activity patterns of red deer in a highly forested and in a patchy forest-agricultural habitat. Acta Silvatica & Lignaria Hungarica 5: 109–118.

[43] Lone K, Loe LE, Meisingset EL, Stamnes I, Mysterud A (2015) An adaptive behavioural response to hunting: surviving male red deer shift habitat at the onset of the hunting season. Anim Behav 102: 127–138.

[44] Sæther B-E, Engen S, Filli F, Aanes R, Schröder W, Andersen R (2002) Stochastic population dynamics of an introduced Swiss population of the ibex. Ecology 83: 3457–3465.

[45] MeteoSwiss (2015) IDAweb. Data portal for teaching and research. Available: http://www.meteoschweiz.admin.ch/web/de/services/datenportal/idaweb.html. Accessed 7 May 2015.

[46] Burnham KP, Anderson DR (2002) Model selection and multimodel inference: a practical information-theoretic approach Springer, New York.

[47] Kass RE, Raftery AE (1995) Bayes factors. J Am Stat Assoc 90: 773–795.

[48] Link WA, Barker RJ (2006) Model weights and the foundations of multimodel inference. Ecology 87: 2626–2635. 17089670 10.1890/0012-9658(2006)87[2626:mwatfo]2.0.co;2

[49] R Core Team (2013) *R*: A language and environment for statistical computing R Foundation for Statistical Computing, Vienna, Austria Available: http://www.R-project.org/.

[50] Mazerolle MJ (2014) AICcmodavg: Model selection and multimodel inference based on (Q)AIC(c). R package version 2.0–1. http://CRAN.R-project.org/package=AICcmodavg.

[51] Darmon G, Calenge C, Loison A, Jullien J-M, Maillard D, Lopez JF (2012) Spatial distribution and habitat selection in coexisting species of mountain ungulates. Ecography 35: 44–53.

[52] Haller RM (2006) Spatial distribution of ungulates in the Swiss National Park–evaluation of survey and analysis methods, and comparison with census results. In: Filli F, Suter W, editors. Nationalpark-Forschung in der Schweiz, 93. Zernez, Switzerland. pp. 45–78.

[53] Haller RM (2011) Integratives Geoinformationsmanagement in der Schutzgebietsforschung Räumliche Genauigkeit als Schlüsselelement des Wissenstransfers. Nationalpark-Forschung in der Schweiz, 95 Haupt-Verlag, Bern, Switzerland.

[54] Sappington JM, Longshore KM, Thompson DB (2007) Quantifying landscape ruggedness for animal habitat analysis: a case study using bighorn sheep in the Mojave desert. J Wildl Manage 71(5): 1419–1426.

[55] Chander G, Markham BL, Helder DL (2009) Summary of current radiometric calibration coefficients for Landsat MSS, TM, ETM+, and EO-1 ALI sensors. Remote Sens Environ 113: 893–903.

[56] Bates D, Maechler M, Bolker B, Walker S (2014) lme4: Linear mixed-effects models using Eigen and S4. R package version 1.1–7. Available: http://CRAN.R-project.org/package=lme4.

[57] Hirzel AH, Hausser J, Chessel D, Perrin N (2002) Ecological-niche factor analysis: how to compute habitat-suitability maps without absence data? Ecology 83(7): 2027–2036.

[58] Schimel D, Stillwell MA, Woodmansee RG (1985) Biogeochemistry of C, N, and P in a Soil Catena of the Shortgrass Steppe. Ecology 66(1): 276–282.

[59] Frank DA (2006) Large herbivores in heterogeneous grassland ecosystems In: Danell K, Bergström R, Duncan P, Pastor J, editors. Large herbivore ecology, ecosystem dynamics and conservation. Cambridge University Press, Cambridge, UK pp. 326–347.

[60] Gordon IJ, Illius AW (1989) Resource partitioning by ungulates on the Isle of Rhum. Oecologia 79: 383–389. 10.1007/BF00384318 23921404

[61] Schweiger AK, Schütz M, Anderwald P, Schaepman ME, Kneubühler M, Haller RM, Risch AC (2015b) Foraging ecology of three sympatric ungulate species: behavioural and resource maps indicate differences between chamois, ibex and red deer. Movement Ecology 3: 6. 26807258 10.1186/s40462-015-0033-x PMC4722786

[62] Belovsky GE (1986) Generalist herbivore foraging and its role in competitive interactions. Am Zool 26: 51–69.

[63] Schweiger AK, Risch AC, Damm A, Kneubühler M, Haller R, Schaepman ME, Schütz M (2015a) Using imaging spectroscopy to predict above-ground plant biomass in alpine grasslands grazed by large ungulates. J Veg Sci 26(1): 175–190.

[64] Macandza VA, Owen-Smith N, Cain JW III (2012) Dynamic spatial partitioning and coexistence among tall grass grazers in an African savanna. Oikos 121: 891–898.

[65] Gottfried M, Pauli H, Futschik A, Akhalkatsi M, Barančok P, Benito Alonso JL, et al (2012) Continent-wide response of mountain vegetation to climate change. Nat Clim Chang 2: 111–115.

[66] Rammig A, Jonas T, Zimmermann NE, Rixen C (2010) Changes in alpine plant growth under future climate conditions. Biogeosciences 7: 2013–2024.

